# Critiquing the response to the Ebola epidemic through a Primary Health Care Approach

**DOI:** 10.1186/s12889-016-3071-4

**Published:** 2016-05-17

**Authors:** Vera Scott, Sarah Crawford-Browne, David Sanders

**Affiliations:** School of Public Health, University of Western Cape, Private Bag X17, Bellville, Cape Town, 7535 South Africa; Primary Health Care Directorate, University of Cape Town, Private Bag, Rondebosch, Cape Town, South Africa

## Abstract

**Background:**

The 2014/2015 West Africa Ebola epidemic has caused the global public health community to engage in difficult self-reflection. First, it must consider the part it played in relation to an important public health question: why did this epidemic take hold and spread in this unprecedented manner? Second, it must use the lessons learnt to answer the subsequent question: what can be done now to prevent further such outbreaks in the future? These questions remain relevant, even as scientists announce that the Guinea Phase III efficacy vaccine trial shows that rVSV-EBOV (Merck, Sharp & Dohme) is highly efficacious in individuals. This is a major breakthrough in the fight against Ebola virus disease (EVD). It does not replace but may be a powerful adjunct to current strategies of EVD management and control.

**Discussion:**

We contribute to the current self-reflection by presenting an analysis using a Primary Health Care (PHC) approach. This approach is appropriate as African countries in the region affected by EVD have recommitted themselves to PHC as a framework for organising health systems and the delivery of health services. The approach suggests that, in an epidemic made complex by weak pre-existing health systems, lack of trust in authorities and mobile populations, a broader approach is required to engage affected communities. In the medium-term health system development with attention to primary level services and community-based programmes to address the major disease burden of malaria, diarrhoeal disease, meningitis, tuberculosis and malnutrition is needed. This requires the development of local management and an investment in human resources for health. Crucially this has to be developed ahead of, and not in parallel with, future outbreaks. In the longer-term a commitment is required to address the underlying social determinants which make these countries so vulnerable, and limit their capacity to respond effectively to, epidemics such as EVD.

**Conclusion:**

The PHC approach offers an insightful critique of the global and regional factors which have compromised the response of health systems in Guinea, Liberia and Sierra Leone as well as suggesting what a strengthened EVD response might involve in the short, medium and long-term.

## Background

The 2014/2015 EVD epidemic evolved into a major humanitarian crisis and, for a period of a few months in late 2014, seemed to be out of control [1]. The global response has been criticised for being “too little, too late” [2, 3] and even irresponsible [4]. Now, as the epidemic ends, various analyses are being offered as to what went wrong in organising an effective response and how a similar situation can be avoided in the future [5–9]. This has been an unusual epidemic in that it occurred in a region of Africa that Ebola Virus Disease (EVD) had not been seen in before [10]. Furthermore, for an EVD outbreak, it was unprecedented in scale, around 65 times larger than the largest previous outbreak: in 2000/2001 in Uganda 425 cases were reported in three months [11]; by 16 September 2015, 28 214 confirmed, probable and suspected cases of EVD with 11 289 deaths were reported in the three most affected countries (Guinea, Liberia and Sierra Leone) [12]. Some argue that EVD is not an ideal candidate for a major epidemic [13] as it has a very high case fatality rate, a low level of infectivity (requiring close contact and exposure to bodily fluid), little evidence of major air-borne spread [14] and, unlike viral influenza for example, those who are infectious show symptoms of major illness. The 2014/2015 West Africa Ebola epidemic has caused the global public health community to engage in difficult self-reflection. First it must consider the part it played in relation to an important public health question: why did this epidemic take hold and spread in this unprecedented manner? Second it must use the lessons learnt to answer the subsequent question: what can be done now to prevent further such outbreaks in the future? These questions remains relevant, even as scientists announce that the Guinea Phase III efficacy vaccine trial shows that rVSV-EBOV is highly efficacious in individuals [15]. This vaccination will now be tested at scale and, if effective, represents a major breakthrough in the fight against EVD. However it should be seen as an adjunct to current strategies of EVD management and control and requires an effective system of delivery to ensure it reaches the populations who need it. A concern emerging in the literature is the role of emergency preparedness in relation to a broader public health response [16–19]. This concern acknowledges that the 2014/2015 epidemic posed unique challenges because of very fragile pre-existing health systems [7, 20, 21]. We contribute to the current self-reflection by presenting an analysis which responds to the key public health questions from the perspective of a Primary Health Care (PHC) approach. We believe that this approach is appropriate as the WHO itself, as well as African countries in the region affected by EVD, have recommitted themselves to PHC as a framework for organising health systems and the delivery of health services in a series of declarations between 2000 and 2008 [22]. The PHC approach offers an insightful critique of the global and regional factors which have led to current fragile health systems in Guinea, Liberia and Sierra Leone as well as suggesting what a strengthened Ebola response might involve in the short, medium and long-term. In this article we describe the transmission and pathogenesis of EVD, the WHO intervention package and its underlying philosophy. We then provide an overview of some of the main factors which, in the current debate, have been argued to contribute to the failure of the traditional approach of outbreak control. Next we present the comprehensive primary health care approach as an alternative philosophical approach, which we use as an analytical framework to generate a further set of insights regarding the importance of acknowledging and addressing the socio-political context of the EVD, and to map out principles of action in health system strengthening.

### Understanding the transmission and pathogenesis of EVD

An understanding of the transmission of EVD is central to informing a health system response. EVD is a zoonosis with animals acting as the reservoir. Humans are infected when they come into contact with the blood, secretions and organs of infected animals such as chimpanzees, gorillas, fruit bats, monkeys, forest antelope and porcupines [21]. All earlier major epidemics have been in Central Africa, in the natural geographic distribution of fruit bats. EVD was first identified in 1976 with two epidemics in Zaire and Sudan; since then there have been more than more than 20 outbreaks in Central Africa which are described by del Rio et al. [23]. With the emergence of EVD in West Africa, it is hypothesized that there has been a major change in the habitat of fruit bats to include this region [10]. Human to human transmission can take place through direct contact (through broken skin or mucous membranes) with the blood, secretions, organs or other bodily fluids of infected people, and indirect contact with environments contaminated with such fluids. Humans are only infectious once they become symptomatic, and the infectivity is low during the first three days of illness [24]. Infectivity increases with time as the person becomes sicker and is especially high around death. Importantly, traditional burial practices are a source of infection [25]. Health workers are particularly at risk when they work in unhygienic and unprotected conditions. Basic protective clothing and gloves to prevent direct contact and maintaining a distance of 1–2 m from infected patients is recommended for infection control [26].

There are no approved drugs to treat the disease. As the disease progresses, the only care possible is supportive; the quality of such care is crucial in determining the outcome. EVD is a gastrointestinal infection which starts as a febrile illness, often with fatigue and myalgia [2]. The predominant symptoms are vomiting and diarrhoea (each is experienced in approximately two-thirds of those infected [20]. The main treatment required is replacement of the lost fluids and management of the electrolyte and acid imbalances that this causes [2]. Bleeding occurs later in the course of the disease, is mainly from the gastrointestinal tract and is present in less than 20 % of those infected [20] and is generally late in the course of disease.

### The WHO intervention package

The World Health Organization (WHO) led the Ebola response from 1 August 2014, including steering the United Nations Mission for Ebola Emergency Response (UNMEER) which was established on 19 September 2014 to coordinate a system-wide United Nation’s response across agencies. The strategy adopted a regional approach but sought to remain specific to the needs of each country. The strategic framework, represented by the acronym STEPP, was to stop the outbreak, treat the infected, ensure essential services, preserve stability and prevent further outbreaks. The Ebola Response Roadmap [27] set out the proposed intervention package, subject to available resources. This included case management of infected patients in Ebola treatment centres (ETCs) which offered isolation, and infection prevention and control activities. Provision was made for referral processes from primary health care facilities to the ETCs and for laboratory-based case diagnosis. Further provision was made for surveillance, contact tracing and monitoring, supervised burials with dedicated expert burial teams and a process of social mobilization to educate on risk reduction and to create support for contact tracing. In intense transmission areas the Roadmap recognised the need for complementary community-based care and community burial teams supported by intensified infection prevention and control with appropriate personal protective equipment. In addition the Roadmap identified the need to ensure essential services in the short-term (such as health services, food, education, water, sanitation and hygiene) recognising that national governments required support in this from non-governmental organisations, United Nations agencies, and humanitarian organizations, and a medium-term investment plan to strengthen health services.

### What philosophy informed the Ebola response roadmap and how successful was it?

The Ebola Response Roadmap was a classic “outbreak control” effort [1]. An outbreak control effort typically involves rapid deployment of technical, medical interventions, and isolation of infectious cases to break the transmission chain. The assumption underlying this approach is that by acting quickly and effectively the outbreak can be brought under control and an epidemic avoided. This end justifies a process in which medical experts take control for the greater good. It is a strategy that has worked in previous outbreaks of EVD in central Africa [20, 21]. These outbreaks were limited in size and geographic spread and the interventions were channelled through the local health care system, with support from international partners. However the rapid growth and complexity of this recent epidemic took organisations like WHO by surprise [28] as it spiralled out of control.

Some believe that the Ebola response contributed to the disintegration of the local health system [1] with uninfected people dying from treatable illnesses such as malaria, respiratory infections and diarrhoea [7]. Primary care facilities were converted to ‘holding centres’ and Ebola Treatment Centres (ETC.); the former were places where patients suspected of having EVD were kept while awaiting transport to take them to the latter. Routine health care services were suspended as the already fragile health systems were overwhelmed by the Ebola response [29]. There is little evidence that hospitalisation during the first few months of the epidemic made a difference to mortality rates in the current epidemic (WHO Ebola Response Team, 2014a). Cure was not the primary objective in managing EVD in Treatment Centres; rather these functioned as part of an elaborate isolation strategy to break transmission. The centres also become part of the problem with nosocomial infection in unhygienic conditions, amplifying the transmission [30]. Health care staff worked in conditions that placed them at risk too, without minimum protective gear such as gloves and many fled [2, 3]. Tragically, a large number of health care staff became infected and died. Some question the value of the model of quarantining patients in treatment centres based on iatrogenic harm [17]. Critically important, the pre-existing health infrastructure was too limited and shortages of health care staff were too severe for the national health systems to respond effectively. Even when massively bolstered by an international donor support-base and resources, treatment beds in Sierra Leone only met 60 % of the need at the peak of the epidemic in November 2014.

The response to the epidemic wrought wider social disruption that threatened local and national economies, food security and social structures. To prevent international spread *The Ebola Response Roadmap* [27] prohibited travel of all cases and contacts, with exit screening for symptoms at international airports, seaports and borders. Affected countries further limited movement between regions using travellers checkpoints and imposed quarantines which varied from stay-at-home days to guarded home confinement [30]. Ten thousand schools were closed, interrupting the schooling of 2 million children [31] and prompting experts to warn that the longer schools remained closed, the greater the risk that children, especially girls, would drop out. This has raised concern that literacy may drop in countries which already have low literacy rates [32] and could have a long-term impact on maternal mortality and child health. In the wake of EVD there has been an increase in household food insecurity due to changes in food production, restricted travel to markets and reduced income potential, with 2.3 to 3 million people estimated to be affected [33].

### Why has the traditional approach of outbreak control failed in this epidemic?

While some postulate a change in the distribution of animal vectors for the emergence of this current EVD epidemic [10], the *escalation* is driven by other factors. There is an emerging consensus that the extraordinary magnitude of the current epidemic is not related to a change in the virus, but due to population density and mobility and other factors [20]. Bausch and Schwarz [10] argue that such large outbreaks almost invariably occur where there is severe poverty and health systems are compromised. In this epidemic, not only was the local, national, and international response too slow but it was complicated by a dysfunctional local health service, unable to act as a conduit for intensified international efforts [1]. All three countries had pre-existing challenges in their health systems with inadequate infrastructure, severe shortages of trained health workers, shortages of basic medicines and very weak health information and disease surveillance systems [9]. In Liberia, Sierra Leone and Guinea in 2013 there were 88 496, 79 365 and 24 096 people per health-centre respectively, compared to 10 320 people per health-centre in nearby Ghana. Instead of the recommended one trained health care worker for every 439 people, there was one health worker for 3 472, 5 319 and 1597people respectively for these three countries. Applying system thinking Agyepong [34] has shown how the EVD outbreak stressed the already compromised health systems, weakening them further in a reinforcing negative cycle. The epidemic was made more complex by occurring in urban, highly-mobile communities moving across the political boundaries of three countries. The ability of the weak national health systems to respond was further compromised by this complexity, and by weak national governance in the three affected countries making the coordination of the health system response more difficult.

In post-conflict Liberia and Sierra Leone, attempts to involve communities were threatened by a lack of trust in authorities [1] which means that top-down implementation models were doomed to failure. Poor treatment outcomes further threatened trust in the newly introduced, alternative health care system, thereby undermining quarantine efforts. The difficulty of involving communities in negotiating appropriate and acceptable treatment and interventions to break transmission chains meant that many infected patients and their families chose to side-step the alienating treatment centres; in Liberia they began to bury their dead secretly to avoid the mandated cremation [35]. Contact tracing suggests that in this epidemic unsafe burial practices accounted for up to 20 % of transmission. The grossly inadequate road infrastructure meant that transporting those infected or suspected of being infected with EVD required long and hazardous journeys consuming scarce resources and increasing the exposure of other patients and health staff to the virus.

### The Comprehensive Primary Health Care approach

Changing theories about the relationship between health and development in the 1970s coupled with concerns about the effectiveness of transplanting medical models of service delivery into developing country settings and interest in alternative community-based models led to the concept of Primary Health Care (PHC) [36, 37] which was ratified in the Alma Ata Declaration [38]. The following definition was put forward:

*“…essential health care based on practical, scientifically sound and socially acceptable methods and technology made universally accessible to individuals and families in the community through their full participation and at a cost that the community and country can afford to maintain… It forms an integral part of the country’s health system of which it is the central function and main focus, and of the social and economic development of the community. It is the first level of contact for individuals, the family and community…bringing health care as close as possible to where people live and work, and constitutes the first element of a continuing health care process”.*

The definition has led to a number of divergent interpretations of PHC [22]. PHC has been interpreted as the first point of contact with the health system or the primary level of care on the one hand, and a broad philosophy or approach to health care on the other. In the latter view, sometimes referred to as the comprehensive PHC approach, it is seen as a strategy for organising health care systems and society to promote health. This perspective has strong socio-political implications, addressing the underlying social determinants of ill-health through intersectoral action, seeking to empower communities, to meet the needs of the most marginalised and to provide comprehensive care with the emphasis on disease prevention and health promotion [39]. It is to this latter perspective that the African Region has re-committed itself [40].

The alternative to the comprehensive PHC approach, the selective approach to PHC [41] proposed focussing on a circumscribed number of diseases with high morbidity and mortality, using largely effective therapeutic or personal preventive interventions. This became the dominant expression of PHC implemented in developing countries [36] and ushered in an area of vertical medical programmes. Baum (2007, p 36) suggests that selective PHC “…*robbed primary health care of its community engagement, broader social change and re-distributive vision and placed it firmly back in the medical framework*”. The importance of investigating the impact of such upstream determinants has been reaffirmed in the last decade, with the findings of the WHO Commission on Social Determinants of Health [42]. Importantly, comprehensive PHC has been recognised as the organising philosophy and framework for African countries [22], as evident in a set of regional declarations such as the *Health-For-All Policy for the 21st Century in the African Region: Agenda 2020* and the *Ouagadougou Declaration on Primary Health Care and Health Systems in Africa: Achieving Better Health for Africa in the New Millennium*. These declarations have an explicit health service focus as well as strong socio-political implications, seeking to respond more equitably, appropriately and effectively to basic health care needs and also address the underlying social, economic and political causes of poor health. The principles of the comprehensive PHC approach include universal accessibility and coverage on the basis of need; comprehensive care with the emphasis on disease prevention and health promotion; community and individual involvement; intersectoral action for health; and appropriate technology and cost-effectiveness in relation to the available resources.

### What does a comprehensive PHC approach offer? Understanding the importance of social determinants in the spread of EVD

A deeper analysis of this EVD outbreak exposes the pathology of the global economic and political system [13]. Despite being resource-rich countries, the populations continue to experience extreme poverty and inequity. It has been suggested that poverty and chronic food shortages in the three countries have led to communities penetrating deeper into the forests to look for food and fuel, potentially exposing them to bats and other animals which are host to the Ebola virus [10]. Further, changes in the local ecology have altered the patterns of the distribution of Ebola’s animal hosts [10]. Deforestation has increased through the growth in the logging industry in Sierra Leone [43] and foreign-owned global agribusiness in Guinea [44] which brings people and wildlife into closer contact with the risk of zoonotic disease [45]. Deforestation also undermines local food production [46]. Even where foreign investment and economic growth is high as in Sierra Leone, transnational corporations have been implicated in tax evasion - see for example [47] - and there are allegations of government corruption in all three countries [9, 48], often serving and exacerbated by foreign interests, as seen in the civil wars in Liberia and Sierra Leone. Civil war itself impoverishes poorer countries [34] and destroys health system infrastructure. For example, in Sierra Leone where the civil war lasted from 1991 to 2002, only 16 % of the health centres were still functional by 1996 [49].

Probing the factors contributing to the long term fragility of health systems of Liberia, Guinea and Sierra Leone reveals insufficient investment despite the fact that these are mineral rich countries. The total health expenditure per capita (USD) in 2013 was $24.80 in Guinea, $44.40 in Liberia and $95.80 in Sierra Leone while the average for Sub-Saharan Africa was $101.30 and globally was $1,047.80. Underinvestment has been linked to a history of exploitation by multinational companies, civil war and corruption [13]. The World Bank and the International Monetary Fund too have played a role through structural adjustment programmes which reduced public spending on welfare and public services [5] and aggravated health worker migration from the pool of critically-stretched trained health workers. More doctors born in Liberia and Sierra Leone work in OECD countries than in their home countries [50]. The international public health community’s focus on disease preparedness has also been implicated in weakening health systems in the region [51]. Fearnley (2015) describes the “emerging diseases worldview” in the 1990s which shaped the development of the then new field of global health, which detracted from building health systems and instead siphoned off resources to focus on disease surveillance and hospital preparedness to quell early epidemics. In the current epidemic EVD has been cast as a global health security threat [7, 23, 30, 52, 53].

An understanding of the social determinants of EVD frames it in a broader socio-political context which needs to be addressed if further outbreaks of this magnitude are to be avoided. The importance of investigating and addressing these factors has been reaffirmed in the last decade, with the findings of the WHO Commission on Social Determinants of Health (World Health Organization & Commission on the Social Determinants of Health, 2008). The global public health community has a responsibility to advocate for and partner with governments to invest in national health systems [13]. Further, to address the underlying poverty and inequity driving the occurrence and spread of EVD, international advocacy is needed to support pro-poor changes in economic and power relations.

### What does a comprehensive PHC approach offer? Principles for action in health system strengthening

In this section we explore the importance and relevance of the principles of comprehensive PHC to the contexts in Guinea, Sierra Leone and Liberia and suggest how they might be applied in a medium and longer-term strategy to address EVD and rebuild health systems in the region. Dubois et al. [9] point out that, in response the Millennium Development Goals (MDGs) and international funding, significant progress in reducing in child and maternal mortality had been made in the region in the decade prior to the 2014/2015 Ebola epidemic. However, they argue that the target-oriented approaches promoted by the MDGs led to vertical funding and ‘narrow bands of attention and progress’ which overlooked the broader agenda of addressing the social determinants driving ill-health and of health system strengthening. This view is supported by others [54] who have noted the ‘fragmented, project-oriented approach to health sector rehabilitation and development, which privileged certain public health problems’ in Sierra Leone after the civil war. There is a call for a more comprehensive approach to health system development. This section does not offer technical recommendations but adds to the small but important literature [9] which is seeking to look beyond the operational issues to underlying systems. We draw on the principles of comprehensive PHC to suggest what such an approach might look like.

#### The re-establishment of the health systems and universal access to comprehensive health care

The comprehensive primary health care approach advocates for access to health care on the basis of equity and social justice. Universal access to health care and prevention has been a challenge in the three most severely affected countries, with their large rural communities and limited health service and transport infrastructure. Access to health care is further reduced by high point-of-treatment costs to the patient that are often unmanageable in subsistence-based agricultural village economies. To control the spread of EVD there is an imperative to ensure that each person is reached to prevent an upsurge in new cases [8]. A comprehensive primary health care approach calls for commitment of skills and funding to supporting the resuscitation and development of a strong primary and community care system - with increased numbers of lower-level and community-based health workers - which gives universal access to prevention and treatment of the common conditions which carry such a high mortality in these countries. Without this there will be an increase in deaths during childbirth, malaria, tuberculosis, human immunovirus/acquired immune deficiency (HIV/AIDS) and acute infectious illnesses [1]. Further, this will ensure that there is an efficient conduit in place for an emergency response to contain future epidemics. However, this would require relinquishing an emergency mind-set [54]. The Free Health Care Initiative, a health reform introduced in 2010 in Sierra Leone, was helpful in coordinating international aid and developing a more comprehensive approach to health system development pre-Ebola, including infrastructure and human resource development [54]. Rebuilding the health system is not a project for heroic, top-down intervention – it is a medium-term project where priority must be given to the process of building the planning and management capacity of the Ministry of Health and district level management to support a strong network of community health workers and primary level facilities. The development of primary care services requires a strong community-based component to ensure adequate coverage of hard-to-reach areas and because complementary home- and community-based interventions are required to be effective [55–57] given the burden of disease. Such a service will be able to identify new cases of EVD and be available to organise itself to deliver new technologies, such as an efficacious EVD vaccine, when they become available.

#### Engagement with communities

The emphasis on breaking transmission to control EVD is an appropriate public health response in the early phase of an epidemic. Up until now this has required early diagnosis and quarantine which, in turn, both require building a relationship of trust between communities and health services [1]. The advent of an effective vaccination still requires engagement with communities who must learn to trust the vaccination, and continues to rely on there being a functional network in the community which can identify the symptoms of EVD and set up a local and effective response. In the current epidemic the WHO Roadmap included social mobilisation [27] but this proved difficult to implement in the midst of the crisis. Fear and suspicion had tragic consequences in some instances; for example riots erupted in Guinea in August 2014 after rumours spread that health workers, who were disinfecting a market, were actually contaminating people; in another incident in Guinea in September 2014 eight members of a team trying to raise awareness about EVD were killed by villagers using machetes and clubs [58]. A key lesson of this EVD outbreak is that it is difficult to engage communities unless there is already a well-developed relationship and a network of health workers who are already accountable to and embedded within communities. A comprehensive primary health care response further advocates for extensive community engagement as a mechanism to give voice to marginalised communities and reduce their vulnerability. Working with existing civil and community structures and local leadership promotes trust-building which is particularly important in settings such as Liberia and Sierra Leone where trust in the government has been broken and where top-down directives are counterproductive.

#### Intersectoral action to address the epidemic

A comprehensive primary health care approach promotes intersectoral action for health, recognising that other sectors such as education often give access to key populations and themselves work towards similar health outcomes, that social determinants can confound health system responses and that social determinants usually underlie the health problems themselves. The importance of intersectoral action is starkly illustrated in relation to food security. In late 2014 the World Food Programme estimated that between 2.3 to 3 million people are food-insecure in the three most affected countries [33], with 0.7 to 1.5 million as a direct or indirect effect of EVD which caused social disruption, affected farming and movement of crops (cassava being the staple) as well as affecting labour markets and people’s livelihoods, meaning that households did not have the money to buy food. In the short-term it meant that there was an urgent need to distribute food to insecure households, a response which was carefully monitored in the later phases of the epidemic [33]. Yet food distribution to outlying communities was thwarted by the poor road infrastructure. Development initiatives are now required to support economies and the re-establishment of local farming practices to reduce vulnerability to future food insecurity. National and local intersectoral action for health therefore needs to be part of the medium term planning involving transport, trade and development sectors.

## Conclusion

A comprehensive primary health care approach adds novel insights into what the global health community might learn from the failure to limit the recent EVD outbreak before it spiralled out of control. The approach suggests that, in an epidemic made complex by weak pre-existing health systems and lack of trust in authorities and mobile populations, a broader approach was required to engage earlier, more actively and more directly with affected communities. In the medium-term health system development with attention to primary level services and community-based programmes to address the major disease burden of malaria, diarrhoeal disease, meningitis, tuberculosis and malnutrition is needed. This requires the development of local management and an investment in human resources for health, including a strong cadre of community workers. Crucially this has to be developed ahead of, and not in parallel with, future outbreaks. In the longer-term a commitment is required to address the underlying social determinants which make these countries so vulnerable, and limit their capacity to respond effectively to, epidemics such as EVD.

## Ethics approval and consent to participate

This article did not involve any primary research with human subjects (including human material or human data). It did not therefore go through an ethics committee. Consent was not an issue.

## Consent for publication

Not applicable.

## Availability of data and materials

All the data supporting analysis is contained within the manuscript.

## Abbreviations

EVD: Ebola virus disease
ETC.: Ebola treatment centres ETCs
HIV/AIDS: Human immunovirus/Acquired immune deficiency
PHC: Primary Health Care
STEPP: Stop the outbreak, treat the infected, ensure essential services, preserve stability and prevent further outbreaks.
UNMEER: United Nations Mission for Ebola Emergency Response
WHO: World Health Organization
